# Effectiveness of physical exam signs for early detection of critical illness in pediatric systemic inflammatory response syndrome

**DOI:** 10.1186/1471-227X-14-24

**Published:** 2014-11-19

**Authors:** Halden F Scott, Aaron J Donoghue, David F Gaieski, Ronald F Marchese, Rakesh D Mistry

**Affiliations:** Section of Emergency Medicine, Department of Pediatrics, Children’s Hospital Colorado and University of Colorado School of Medicine, 13123 East 16th Avenue, B251, Aurora, CO 80045 USA; Section of Emergency Medicine, Department of Pediatrics, The Children’s Hospital of Philadelphia, 34th Street & Civic Center Boulevard, Philadelphia, PA 19104 USA; Department of Anesthesia & Critical Care, The Children’s Hospital of Philadelphia, 3400 Spruce Street, Philadelphia, PA 19104 USA; Department of Emergency Medicine, The Hospital of the University of Pennsylvania, 3400 Spruce Street, Philadelphia, PA 19104 USA; The Center for Resuscitation Science, The Hospital of the University of Pennsylvania, 3400 Spruce Street, Philadelphia, PA 19104 USA

**Keywords:** Sepsis, Systemic inflammatory response syndrome, Pediatrics, Physical examination, Emergency medicine

## Abstract

**Background:**

Early detection of compensated pediatric septic shock requires diagnostic tests that are sensitive and specific. Four physical exam signs are recommended for detecting pediatric septic shock prior to hypotension (cold extremities, mental status, capillary refill, peripheral pulse quality); this study tested their ability to detect patients who develop organ dysfunction among a cohort of undifferentiated pediatric systemic inflammatory response syndrome patients.

**Methods:**

A prospective cohort of 239 pediatric emergency department patients <19 years with fever and tachycardia and undergoing phlebotomy were enrolled. Physicians recorded initial physical exams on a standardized form. Abstraction of the medical record determined outcomes including organ dysfunction, intensive care unit stay, serious bacterial infection, and therapies.

**Results:**

Organ dysfunction occurred in 13/239 (5.4%) patients. Presence of at least one sign was significantly associated with organ dysfunction (Relative Risk: 2.71, 95% CI: 1.05–6.99), and presence of at least two signs had a Relative Risk = 4.98 (95% CI: 1.82–13.58). The sensitivity of exam findings ranged from 8–54%, specificity from 84–98%. Signs were associated with increased risk of intensive care and fluid bolus, but not with serious bacterial infection, intravenous antibiotics or admission. Altered mental status and peripheral pulse quality were significantly associated with organ dysfunction, while abnormal capillary refill time and presence of cold, mottled extremities were not.

**Conclusions:**

Certain recommended physical exam signs were associated with increased risk of organ dysfunction, a rare outcome in this undifferentiated pediatric population with fever and tachycardia. Sensitivity was low, while specificity was high. Additional research into optimally sensitive and specific diagnostic strategies is needed.

**Electronic supplementary material:**

The online version of this article (doi:10.1186/1471-227X-14-24) contains supplementary material, which is available to authorized users.

## Background

Severe sepsis affects >72,000 children yearly in the US, with mortality estimates from 10–20% [1, 2]. Adherence to time-sensitive pediatric sepsis treatment guidelines reduces mortality; [3] however, delayed recognition often leads to deviation from these guidelines [4]. Contributing to the challenge of recognition of pediatric sepsis are the large numbers of febrile children with self-limited illness who present to emergency departments for treatment [5]. Therefore, identifying optimal strategies for early identification of pediatric sepsis, particularly of patients in a state of compensated shock, is paramount.

American College of Critical Care Medicine (ACCM) guidelines recommend specific physical examination findings for identification of pediatric septic shock requiring resuscitation; this approach has been adopted into pediatric emergency department (ED) triage strategies [6–8]. In addition, the pediatric guidelines recommend against the use of laboratory tests to identify septic shock. However, these specific physical examination findings have not been prospectively evaluated for their effectiveness in diagnosing septic shock in patients outside of the intensive care setting.

Current ACCM guidelines for early recognition of septic shock recommend assessment of mental status, capillary refill, peripheral pulse quality, cold/mottled extremities, urine output, and hypotension [6]. At initial presentation, urine output is difficult to quantify, and hypotension defines decompensated shock; therefore four of the recommended physical examination signs remain useful for detection of compensated sepsis in the ED. We tested the effectiveness of these four parameters, or Clinical Recognition Signs (CRS), in predicting severe illness in pediatric ED patients with potential early sepsis.

## Methods

This was a planned subanalysis of a prospective cohort study testing lactate levels for diagnosis of sepsis, and was conducted in the ED of a free-standing children’s hospital. Inclusion criteria were: age <19 years; triage heart rate and temperature consistent with systemic inflammatory response syndrome (SIRS) definitions [9] [Additional file 1]; phlebotomy performed in ED; and enrollment preceding or ≤15 minutes after intravenous fluid initiation. Patients transferred from another institution, or with an inborn error of metabolism were excluded. This hospital did not have a sepsis screening or treatment protocol in place at the time of the study. The Children’s Hospital of Philadelphia Institutional Review Board approved this study, with a full waiver of informed consent due to demonstrated barriers in obtaining timely and informed consent from critically ill patients and families prior to receipt of treatments.

Pediatric emergency medicine (PEM) fellows and attendings recorded CRS at the time of their first examination using multiple choice categorical responses that corresponded to the severity of each finding on a data collection form [Additional file 2]. In order to capture real-world effectiveness, physicians were not explicitly trained in physical examination for CRS. All study physicians were board-certified in pediatrics and/or pediatric emergency medicine and >90% of their active clinical practice was in pediatric emergency medicine. When possible, a second PEM physician examined the subject, and independently completed a data collection form to determine inter-rater reliability. Details of the ED visit and subsequent hospitalization, obtained from the medical record, were recorded after discharge on a data collection form.

All discharged patients underwent review of subsequent visits in the inpatient and outpatient electronic medical record within 90 days of the index visit. Evidence of severe sepsis that developed within 3 days of ED presentation, or new problems that developed after the primary visit potentially attributable to a shock episode treated elsewhere, were recorded. For patients without follow up visits in the hospital system, the Social Security Death Index was reviewed and deaths recorded.

The main predictor variables were abnormal CRS. Each CRS was analyzed as a binary variable (normal/abnormal) and categorical, and presence of ≥1 and ≥2 signs was analyzed. Clinically relevant and frequently referenced combinations of CRS were evaluated. A patient was classified as having cold shock when weak peripheral pulses and prolonged capillary refill were present, warm shock when bounding pulses and flash capillary refill were present, and World Health Organization-Emergency Triage and Treatment (WHO-ETAT) shock when cold extremities, capillary refill >3 seconds and weak peripheral pulses were present. The primary outcome measure was presence of organ dysfunction (OD) within 24 hours of ED triage, as defined by International Pediatric Sepsis Consensus Conference definitions [Additional file 3] [9]. Secondary outcomes included ED disposition, intravenous antibiotic administration in the ED, intravenous fluid bolus administration in the ED, and serious bacterial infection (SBI). SBI was defined as a pathogen-positive culture from blood, urine, or cerebrospinal fluid, or definite infiltrate by pediatric attending radiologist interpretation of chest radiograph.

Sample size was calculated for the parent study of serum lactate levels in pediatric SIRS, and enrollment carried out over 13 months to satisfy that sample size [10]. Study data were summarized using standard descriptive statistics. The magnitude of associations between categorical variables and outcomes were described using relative risks. Logistic regression analysis was performed with CRS found to be significant in univariate analysis; receipt of intravenous antibiotics and receipt of 40 ml/kg of isotonic fluid were covariables. Test characteristics (sensitivity, specificity, likelihood ratios) were calculated. Relative risks and likelihood ratios whose 95% confidence intervals did not include one were considered statistically significant. The incidence and test characteristics of cold shock, warm shock and WHO-ETAT shock were evaluated. The kappa statistic or percent concordance when appropriate, were calculated as measures of inter-rater reliability.

## Results

239 eligible patients were enrolled and their characteristics are shown in Table 1. Over two-thirds of subjects (67.8%) had no CRS; 64 (26.7%) subjects had one CRS; ten (4.2%) subjects had two CRS, and three (1.3%) subjects had three CRS. No patient displayed all four abnormal CRS. Organ dysfunction within 24 hours developed in 5.4% subjects. 76.6% of patients were hospitalized; 8.0% were admitted to the intensive care unit (ICU), and no patients died.

**Table 1:** **Subject characteristics**

Characteristic	N	%
**Age**		
Infants (<3 mo)	19	7.9
Toddlers (3 mo to <2 yr)	68	28.5
School-age (2 yr to <13 yr)	131	54.8
Adolescents (13 yr to <19 yr)	21	8.8
**Sex**		
Male	128	53.6
**Race/ethnicity**		
African American	120	50.2
Caucasian	73	30.5
Asian/Hispanic/Other	46	19.3
**ED disposition**		
Admission to Ward	164	68.6
Discharged to Home	56	23.4
Intensive Care Unit	19	8.0
**Serious bacterial infection**	53	22.2
Pneumonia	33	13.8
Urinary Tract Infection	14	5.9
Bacteremia	9	3.8
**Intravenous antibiotics**	149	62.3
**Fluid bolus (≥20 ml/kg)**	116	48.5
**IV antibiotics + ≥40 mL/kg IV fluid**	17	7.1
**Organ dysfunction within 24 hours**	13	5.4
Organ Dysfunction In ED	9	3.7
Organ Dysfunction After ED	4	1.7
**Hypotension within 24 hours**	5	2.1
Hypotension in Triage	0	0
Hypotension in ED	2	0.8
Hypotension after ED	3	1.3

The presence of ≥1 CRS was significantly associated with OD within 24 hours (RR: 2.71, 95% CI 1.05–6.99), and presence of ≥2 CRS carried greater risk of OD (RR: 4.98, 95% CI: 1.82–13.58). In analysis of each sign as normal/abnormal, altered mental status and altered peripheral pulse quality were significantly associated with risk of OD, while abnormal capillary refill time and cold, mottled extremities were not [Table 2]. The negative predictive value of exam findings ranged from 94–97%, and positive predictive value ranged from 3–25% [Table 2]. When altered mental status and altered peripheral pulses were analyzed with logistic regression with intravenous antibiotics and receipt of at least 40 ml/kg of isotonic fluid as covariables, only altered mental status remained significantly associated with organ dysfunction, with an odds ratio of 3.84 (1.083–13.62).

**Table 2:** **Sepsis clinical recognition signs present in ED as predictors of organ dysfunction within 24 hours**

Individual predictor	Prevalence n (%)	Sensitivity	Specificity	Negative predictive value	Positive predictive value	Positive likelihood ratio	Concordance**
Altered Mental Status	43 (18%)	0.54 (0.29–0.77)	0.84 (0.78–0.88)	0.97 (0.93–0.99)	0.16 (0.07–0.30)	3.3* (1.8–5.9)	76%
Abnormal Capillary Refill	36 (15%)	0.08 (0.01–0.33)	0.85 (0.79–0.89)	0.94 (0.90–0.97)	0.03 (0.001–0.15)	0.5 (0.1–3.4)	96%
Abnormal Peripheral Pulses	8 (3%)	0.15 (0.04–0.42)	0.97 (0.94–0.99)	0.95 (0.92–0.98)	0.25 (0.03–0.65)	5.8* (1.2–26.0)	92%
Cold/Mottled Extremities	5 (2%)	0.08 (0.01–0.33)	0.98 (0.95–0.99)	0.95 (0.91–0.97)	0.20 (0.01–0.72)	4.3 (0.5–36.2)	100%
**Number of predictors**							
≥1	77 (32.2%)	0.62 (0.32–0.86)	0.69 (0.63–0.75)	0.97 (0.93–0.99)	0.10 (0.05–0.19)	2.0* (1.3–3.2)	72%
≥2	15 (6.3%)	0.23 (0.05–0.54)	0.95 (0.90–0.97)	0.96 (0.92–0.98)	0.20 (0.04–0.48)	4.4* (1.4–13.5)	72%

*Statistically significant associations.

**Based on 25 patients with two independent assessments.

The presence of ≥1 or ≥2 CRS was associated with an increased likelihood of ICU admission. The presence of ≥2 CRS was associated with an increased likelihood of receiving a fluid bolus [Table 3]. CRS were not associated with intravenous antibiotic administration, SBI, or admission. Mean time from triage to antibiotic in the overall study population was 3.40 ± 1.49 hours; time in patients with ≥1 CRS was 3.34 ± 0.44 hours, and in patients with ≥2 CRS it was 3.05 ± 0.69 hours.

**Table 3:** **Relative risk of clinical outcomes in patients with clinical recognition signs present on initial physical examination**

	SBI (95% CI)	Admission (95% CI)	ICU in 24 hours (95% CI)	ED bolus (95% CI)	ED antibiotics (95% CI)
≥1 Clinical Recognition Sign	0.83 (0.49–1.41)	0.95 (.81–1.11)	2.57* (1.11–5.95)	1.19 (0.92–1.56)	1.00 (0.81–1.23)
≥2 Clinical Recognition Signs	0.29 (0.04–1.94)	.96 (0.70–1.31)	3.73* (1.43–9.78)	1.56* (1.12–2.19)	0.74 (0.42–1.28)

*Statistically significant association.

Within each of the four CRS, each abnormal finding was independently evaluated for association with the outcome of organ dysfunction. The abnormal mental status category of “agitation” (RR: 5.75, 95% CI: 2.07–16.0) and the pulse abnormality category of “weak/thready” (RR: 9.63, 95% CI: 2.17–42.77) were the only specific findings significantly associated with organ dysfunction. Few patients displayed the combination of findings specific to cold shock (0), warm shock (1), or WHO shock (0) that had been planned for analysis. The patient displaying characteristics of warm shock did not develop organ dysfunction.

PEM attendings completed 79.9% of assessments; PEM fellows completed 12.1%, and general pediatricians completed 8.0%. Exams were completed before institution of intravenous therapies for nearly all subjects (95.8%). Of the 10 (4.2%) exams for which the exams were performed after therapy had been initiated, all exams were performed within 15 minutes of the start of intravenous therapy as specified in the inclusion criteria. Subjects had received a mean of 9 ml/kg of fluid prior to examination for CRS. Two patients were examined within 15 minutes after the start of an antibiotic infusion; no patients were examined after vasoactive agents. None of the patients who were enrolled after the start of intravenous therapy had either the predictor, CRS, or the outcomes of organ dysfunction or serious bacterial infection. Kappa as a measure of inter-rater reliability could not be calculated in several categories where only normal findings were present, therefore percent concordance rather than the kappa statistic is presented in Table 2.

Review of the medical records of the 56 patients discharged primarily from the ED revealed that 21 (37.5%) subjects had made additional visits in the healthcare system, although none were within 72 hours for progression of illness. The additional visits demonstrated that no patients were hospitalized for progression of primary process at the index visit, and no records of additional new medical problems attributable to sepsis or death. Search of the Social Security Death Index for the 35 subjects without further visits in our system yielded no reported deaths.

## Discussion

Previous studies have not evaluated all four CRS at the time of presentation to care as predictors in children with SIRS, though these findings are consistent with prior studies of individual predictors. In mixed-severity populations of children, capillary refill time has been demonstrated to have limited value as a predictor of illness severity in infectious illness [11, 12]. However, a large study of patients referred for specialty transport to children’s hospitals found that abnormal capillary refill times were associated with increased mortality [3]. This likely reflects differing effectiveness of abnormal capillary refill time for determining risk of organ dysfunction in an undifferentiated low-risk population, compared to its ability to stratify risk of death in a high risk, critically-ill population. Additionally, capillary refill times have been demonstrated to be significantly altered by ambient temperature, thus the capillary refill time measured by an ED physician early in a patient’s course may be artificially slow due to outside temperature or undressing a child, potentially accounting for its lack of association with outcome in this study [13].

To have SIRS, a patient must display two out of four criteria. This study only evaluated patients with SIRS by two criteria that are readily available in triage, heart rate and temperature, and thus these results would not necessarily be the same in patients fulfilling different SIRS criteria. Patients with SIRS and phlebotomy during their ED stay were enrolled in order to increase the severity of illness in the study cohort compared to the general SIRS population, resulting in a study population with a 75% admission rate. These results cannot be extrapolated to all ED patients with SIRS, in which a lower pre-test probability for severe illness would likely make the positive predictive value of these tests lower. In addition, in order to study effectiveness, physical examination was performed by uncoached PEM physicians. It is possible that with additional training and standardization, the examination findings would display improved test characteristics; nonetheless, these findings represent their effectiveness when used by physicians who are highly trained and practice in the setting in which these examination techniques are frequently used. The study was not powered to estimate sensitivity with narrow confidence intervals, which would have required thousands of patients given the low prevalence of organ dysfunction; nonetheless the likelihood ratios from our study sample and analysis are informative. This was by intention a snapshot study of these physical exam characteristics. The conclusions limited to these four exam parameters performed once at the outset of ED care.

There are limitations to the study. The most significant limitation was the small number of patients with the primary outcome, organ dysfunction. Sample size was calculated for the parent study of lactate in the ED, and in these calculations a higher percentage of patients with organ dysfunction than was actually seen had been estimated prior to the study. With little literature to guide estimates of organ dysfunction in an undifferentiated pediatric SIRS population, adult and pediatric critical care data were used for these estimates. The low frequency of our outcome influences the wide confidence intervals of the results, as well as the low positive predictive value of CRS. Although not intended to determine the prevalence of organ dysfunction in pediatric SIRS, it is likely that the low frequency of this outcome in the study population accurately reflects of the needle-in-a-haystack challenge of detecting severe sepsis among all children with fever and tachycardia. Additionally, although all patients enrolled demonstrated fever and tachycardia, suggesting infection as a contributing problem, it is possible that organ dysfunction due to other, non-sepsis diagnoses may have confounded the data.

Children were enrolled before or within 15 minutes of the start of intravenous therapy; before therapy for >95% of patients. Because of the challenges of screening and enrolling patients in emergency research, and the near-simultaneous physical examination and therapeutic decision-making that an emergency physician carries out, this window of time was considered a practical window in which to enroll patients, in which intravenous therapy might be expected to minimally alter examination findings. Although this could have potentially influenced results, none of the patients enrolled after the start of intravenous therapy had the predictor, CRS, or the outcome, organ dysfunction, so if it biased results, it would have only served to falsely improve test characteristics of CRS. The study population was approximately half African-American, 30% Caucasian, and 20% other. This may limit its generalizability to different populations, particularly if the test characteristics and reproducibility of physical exam findings varies by race.

Because ED treatment was not standardized, it is possible that if patients with CRS received more aggressive treatment, it would have prevented them from developing the primary outcome of organ dysfunction. However, there was no difference in administration of fluid or antibiotics, aside from the patients with at least two CRS who were more likely to receive an intravenous fluid bolus. Mean time to antibiotic was slightly faster in patients with CRS, however all times were over 3 hours from triage, and the only interval that has been demonstrated to differentially improve outcomes in ED sepsis patients is a time <1 hour [14]. The lack of protocol and time to antibiotics do not represent ideal sepsis care, but are consistent with the majority of emergency department pediatric sepsis care described in prior descriptive studies [4, 15, 16].

Physical examination remains medicine’s well-honed technique for assessment of patient condition. Experienced clinicians frequently determine that a patient appears “septic” based on a clinical impression that considers exam findings and patient characteristics. However, with evidence that life-saving resuscitation begins in the first minutes of ED care, all diagnostic modalities deserve critical evaluation to determine those that accurately risk-stratify patients from the moment of presentation, including the specific characteristics currently recommended for this purpose. Standardization of assessing these physical exam parameters, the use of additional historical and physical findings, traditional laboratory parameters, novel biomarkers, and non-invasive devices for measuring cardiac output and perfusion are all avenues for research with potential to improve diagnosis of early pediatric sepsis.

Timely sepsis resuscitation is life saving, yet at the same time it requires many resources: personnel at the bedside, placement of intravenous peripheral or central catheters, rapid procurement and administration of intravenous fluid boluses, antibiotics and potentially additional medications. Unnecessary aggressive resuscitation diverts resources from other patients, increases costs, and potentially induces medical and psychological harm. With serious negative consequences to both the under-diagnosis and over-diagnosis of sepsis requiring resuscitation, diagnostic strategies that are both sensitive and specific must be sought.

## Conclusions

This investigation evaluated physical examination CRS for early diagnosis of pediatric sepsis in the ED. It demonstrated that the presence of ≥1 or ≥2 CRS was significantly associated with OD and ICU admission, but not associated with ED antibiotic administration, SBI, or admission. Abnormal peripheral pulse quality and altered mental status were significantly associated with development of OD, while abnormal capillary refill and presence of cold, mottled extremities were not.

This study has implications for sepsis screening protocols. The high negative predictive values of CRS make their absence reassuring that a patient is unlikely to progress to organ dysfunction. However, the association of one CRS with organ dysfunction was modest. Although the clinical recognition signs recommended for diagnosis of pediatric sepsis are specific, they lack the positive predictive value that would be ideal for a test in sepsis, and research should continue to determine tests that diagnose children with compensated septic shock with both specificity and sensitivity.

## Electronic supplementary material

Additional file 1: **SIRS Definitions.** This file details age-specific SIRS parameters, as defined in the reference cited. (PDF 81 KB)

Additional file 2: **Questionnaire Completed by Physicians in the Emergency Department Describing their Initial Physical Examination of the Patient.** This file is a replica of the study data collection form used to record the physical exam. (PDF 68 KB)

Additional file 3: **Study Organ Dysfunction Definitions, adapted from IPSCC definitions.** This file contains the definitions used for assessing presence of the primary outcome, organ dysfunction. (PDF 62 KB)

## Abbreviations

ACCM: American College of Critical Care Medicine
CRS: Clinical recognition sign
ED: Emergency Department
ICU: Intensive care unit
OD: Organ dysfunction
PEM: Pediatric emergency medicine
SBI: Serious bacterial infection
SIRS: Systemic inflammatory response syndrome.
