# Supplementary material for: Effectiveness of physical exam signs for early detection of critical illness in pediatric systemic inflammatory response syndrome
Source: BMC Emerg Med. 2014 Nov 19;14:24. doi: 10.1186/1471-227X-14-24 (PMC4289256; doi:10.1186/1471-227X-14-24)
Supplement: Supplementary file 1 — Additional file 1: SIRS Definitions. This file details age-specific SIRS parameters, as defined in the reference cited. (PDF 81 KB) [file 12873_2014_216_MOESM1_ESM.pdf]

Additional File 1. Pediatric SIRS criteria. Requires two of four criteria, one of which must be Temperature>38.5 or <36 or alteration in leukocyte count. In this study, only heart rate and temperature SIRS abnormalities were used for inclusion.[1]

AGE	Tachycardia (beats/min)	Bradycardia (beats/min)	Respiratory Rate (breaths/min)	Leukocyte Count (Leukocytesx10^3/mm)
0 Day to < 1 Week	>180	<100	>50	>34
1 Week to < 1 Month	>180	< 100	>40	>19.5 or <5
1 Month to <2 Years	> 180	< 90	>34	>17.5 or <5
2 Years to <6 Years	> 140	NA	>22	>15.5 or <6
6 Years to < 13 Years	> 130	NA	>18	>13.5 or <4.5
13 Years to < 18 Years	> 110	NA	>14	>11 or <4.5

1. Goldstein B, Giroir B, Randolph A, International Consensus Conference on Pediatric S: **International pediatric sepsis consensus conference: definitions for sepsis and organ dysfunction in pediatrics**. *Pediatric critical care medicine : a journal of the Society of Critical Care Medicine and the World Federation of Pediatric Intensive and Critical Care Societies* 2005, **6**(1):2-8.
