# Supplementary material for: Effectiveness of physical exam signs for early detection of critical illness in pediatric systemic inflammatory response syndrome
Source: BMC Emerg Med. 2014 Nov 19;14:24. doi: 10.1186/1471-227X-14-24 (PMC4289256; doi:10.1186/1471-227X-14-24)
Supplement: Supplementary file 2 — Additional file 2: Questionnaire Completed by Physicians in the Emergency Department Describing their Initial Physical Examination of the Patient. This file is a replica of the study data collection form used to record the physical exam. (PDF 68 KB) [file 12873_2014_216_MOESM2_ESM.pdf]

Additional File 2: Questionnaire Completed by Physicians in the Emergency Department Describing their Initial Physical Examination of the Patient

Mental Status:

ALERT or upset/anxious in a way that seems normal to you
AGITATED or IRRITABLE
LETHARGIC or SOMNOLENT or CONFUSED
RESPONSIVE TO PAINFUL STIMULI ONLY
UNRESPONSIVE

Capillary Refill Time:

<2 SECONDS
FLASH (appears abnormally rapid to you)
3 SECONDS
4 OR MORE SECONDS

Peripheral Pulses:

NORMAL
BOUNDING (feels abnormally strong to you)
WEAK / THREADY
ABSENT

Are the patient’s extremities COLD or MOTTLED? YES / NO
