# Supplementary material for: Effectiveness of physical exam signs for early detection of critical illness in pediatric systemic inflammatory response syndrome
Source: BMC Emerg Med. 2014 Nov 19;14:24. doi: 10.1186/1471-227X-14-24 (PMC4289256; doi:10.1186/1471-227X-14-24)
Supplement: Supplementary file 3 — Additional file 3: Study Organ Dysfunction Definitions, adapted from IPSCC definitions. This file contains the definitions used for assessing presence of the primary outcome, organ dysfunction. (PDF 62 KB) [file 12873_2014_216_MOESM3_ESM.pdf]

Additional File 3: Study Organ Dysfunction Definitions, adapted from IPSCC definitions[1]

Cardiovascular Dysfunction (any of the following):

- Despite isotonic intravenous bolus ≥40 ml/kg
  - Systolic Blood Pressure <5% for age or
  - Need for vasoactive drug (dopamine > 5µg/kg/min, or dobutamine, epinephrine, norepinephrine)
- Capillary refill >5 seconds
- Urine output<0.5 cc/kg/hour

Respiratory Dysfunction (any of the following):

- PaO2/FIO2 <300 in absence of cyanotic heart disease or preexisting lung disease
- PaCO2 > 65 torr or 20 mm Hg over baseline
- Proven need for >50% FIO2 to maintain saturation ≥92%

Neurologic Dysfunction (any of the following):

- Glasgow Coma Scale ≤11 or acute change ≥3 points below abnormal baseline

Hematologic Dysfunction (any of the following):

- Platelets <80,000 or decline of 50% from highest value over past 3 days in patients with baseline low platelets
- International Normalized Ratio >2

Renal Dysfunction:

- Creatinine ≥2 times upper limit for age or 2-fold increase in baseline creatinine in patients with baseline elevations in creatinine

Hepatic Dysfunction (any of the following):

- Total bilirubin ≥4 (not applicable to newborn)
- Alanine transaminase (ALT) 2 times upper limit of normal for age or 2-fold increase in baseline abnormal ALT

[1]*Goldstein B, Giroir B, Randolph A. International pediatric sepsis consensus conference: definitions for sepsis and organ dysfunction in pediatrics. Pediatr Crit Care Med 2005;6:2-8.*
